# Reaction and diffusion thermodynamics explain optimal temperatures of biochemical reactions

**DOI:** 10.1038/s41598-018-28833-9

**Published:** 2018-07-23

**Authors:** Mark E. Ritchie

**Affiliations:** Department of Biology, Syracuse University, 107 College Place, Syracuse, NY 13244 USA

## Abstract

Ubiquitous declines in biochemical reaction rates above optimal temperatures (*T*_*opt*_) are normally attributed to enzyme state changes, but such mechanisms appear inadequate to explain pervasive *T*_*op*t_ well below enzyme deactivation temperatures (*T*_*den*_). Here, a meta-analysis of 92 experimental studies shows that product formation responds twice as strongly to increased temperature than diffusion or transport. This response difference has multiple consequences for biochemical reactions, such as potential shifts in the factors limiting reactions as temperature increases and reaction-diffusion dynamics that predict potential product inhibition and limitation of the reaction by entropy production at temperatures below *T*_*den*_. Maximizing entropy production by the reaction predicts *T*_*opt*_ that depend on enzyme concentration and efficiency as well as reaction favorability, which are patterns not predicted by mechanisms of enzyme state change. However, these predictions are strongly supported by patterns in a meta-analysis of 121 enzyme kinetic studies. Consequently, reaction-diffusion thermodynamics and entropy production may constrain organism performance at higher temperatures, yielding temperature optima of life that may depend on reaction characteristics and environmental features rather than just enzyme state changes.

## Introduction

Understanding the response of organisms to changes in temperature is fundamental in the life sciences. The response of organism performance to increases in temperature ultimately depends on how underlying enzyme-catalyzed biochemical reaction rates change with higher temperature. Rates of relevant biochemical reactions typically increase exponentially with increased temperature at low temperature ranges^1–3^, but slow and then decline above optimal temperatures (*T*_*opt*_). Declines are generally attributed to state changes in enzymes resulting from different mechanisms, including enzyme denaturation, changes in the charge distribution at the enzyme active site^4^, changes in heat capacity of enzyme-bound intermediates^5–7^, differential temperature sensitivity of substrate-enzyme and enzyme-product transitions^8^ or some combination of these^9^.

Despite its entrenchment as a hypothesis, molecular state changes cannot account well for many commonly observed features of *T*_*opt*_. For example, *T*_*op*t_ of reactions measured *in vivo* are often more than 20 °C below reported *in vitro* enzyme de-activation or denaturation temperatures, *T*_*den*_^4,5,10,11^ and enzyme activities are often sustained well above reported *in vitro T*_*den*_ in the presence of co-solvents or heat shock proteins^7,12,13^. *T*_*opt*_ may depend on reaction characteristics as much or more than molecular state changes, as *T*_*opt*_ often changes by 10–20 °C in response to changes in enzyme efficiency^14,15^ or concentration^16,17^. There is currently no rigorous theoretical explanation for why *T*_*opt*_ would change with these reaction characteristics.

An alternative mechanism of temperature dependence in biochemical reactions, in which no state changes in enzymes or their state transitions need occur, is if temperature sensitivity of product formation at reaction sites is different from that of diffusion or transport of substrates, products and heat. Friction generated by collisions of diffusing molecules^6,18^ could dramatically reduce the temperature sensitivity of diffusion or transport of molecules^19^. If so, failure to dissipate accumulated products and/or heat from reaction sites at higher temperatures may lead to faster reverse reactions^20–23^, or increases in internal entropy^21,24,25^ that translate into reduced reaction rates and biological performance. Here I combine unprecedented meta-analyses with new theoretical explorations to show that (1) diffusion or transport exhibits lower temperature sensitivity compared to that of product formation in enzyme-catalyzed reactions, and (2) such a difference can lead to thermodynamic limitation of reactions at temperatures below *T*_*den*_, such that *T*_*opt*_ depends on reaction, enzyme, and cell environmental characteristics. These patterns are not predicted by molecular state change mechanisms.

Maintaining irreversible substrate-to-product flows is important to sustaining cell metabolic infrastructure and potential^20,21,25^, as cells or cell structures and enzymes need to continue to persist despite heat-generating reactions. This requires reactions to confer little change on catalytic enzymes, membranes and other respiratory infrastructure and for cells to dissipate products and heat away from reaction sites^20,21,26^. Accounting for reaction products and heat simultaneously^25^ yields a constraint on reaction rate. This is evident from analysis of the familiar change in Gibbs free energy, Δ*G*, as a function of change in enthalpy Δ*H*, change in entropy Δ*S*, and temperature *T*(°K) for a chemical conversion under no change in pressure or volume, Δ*G* = Δ*H*−Δ*ST*. These changes can be expressed as a sum of rates:

1 $${\rm{\Delta }}G\,{k}_{S}=dH/dt{{-}}TdS/dt,$$

where *k*_*S*_ is a reaction constant (time^−1^ or mol.time^−1^), d*H*/d*t* is the rate of change in enthalpy in the system and d*S*/d*t* the rate of change in entropy due to chemical work. d*H*/d*t* can be redefined as the heat loss *Q* and d*S*/d*t* is the entropy production from chemical work, σ_C_. From the Van’t Hoff equation, Δ*G* = *RT* ln(*a*) where *R* is the gas constant and *a* is the chemical activity, and thus the entropy production-limited reaction constant is

2 $${k}_{S}=(-Q/T+{{\rm{\sigma }}}_{{\rm{C}}})/R\,\mathrm{ln}(1/a),$$

where *−Q/T* is the (positive) rate of entropy production in the surroundings due to heat dissipation. Equation (2) clearly shows that the net forward reaction constant *k* increases with higher entropy production relative to free energy. Note that tracking entropy changes allows for both the changes in the number of molecular states (internal entropy), as reflected in concentration of reactants and products, and dissipation of heat and products to the surroundings (external entropy) to be considered simultaneously. In addition, Equation (2) does not strictly apply only to exothermic reactions, as net input of heat (positive *Q*) can produce positive *k* even when 1/*a* < 1, as would be expected for an endothermic reaction^27,28^.

Recognzing that the reaction site is held potentially far from equilibrium by both the delivery of substrate (reactants) to and products from reaction sites, a reaction-diffusion description^24,25^ of the reaction is appropriate. The chemical activity *a* is the ratio of products to substrates relative to the ratio at equilibrium, *K*_*eq*_. Consequently, a reaction-diffusion model of an idealized enzyme-catalyzed reaction was constructed to account for potential differences in temperature sensitivity of diffusion coefficients and reaction constants. This model yields a predicted optimal temperature that maximizes total entropy production outside the reaction site from movement (diffusion and transport), heat dissipation and chemical work^20,24,25,29^. This optimal temperature turns out to depend on a number of reaction characteristics and environmental conditions, unlike optimal temperatures predicted by molecular state change hypotheses.

## Results and Discussion

Reaction rates and diffusion and transport are commonly found to increase exponentially with temperature. This is captured in Boltzmann temperature dependence for reaction constants, *k*, and diffusion coefficient *D* in viscous fluids^23,29^ (Equation (3))

3 $$D={d}_{0}{e}^{-{{\rm{E}}}_{{\rm{D}}}/{\rm{RT}}};\,k={k}_{0}{e}^{-{{\rm{E}}}_{{\rm{Z}}}/{\rm{RT}}}$$

where *d*_0_ and *k*_0_ are constants. *R* is the gas constant, and *E*_*D*_ and *E*_*Z*_ are activation energies for diffusion/transport and product formation, respectively.

**Figure 1:**
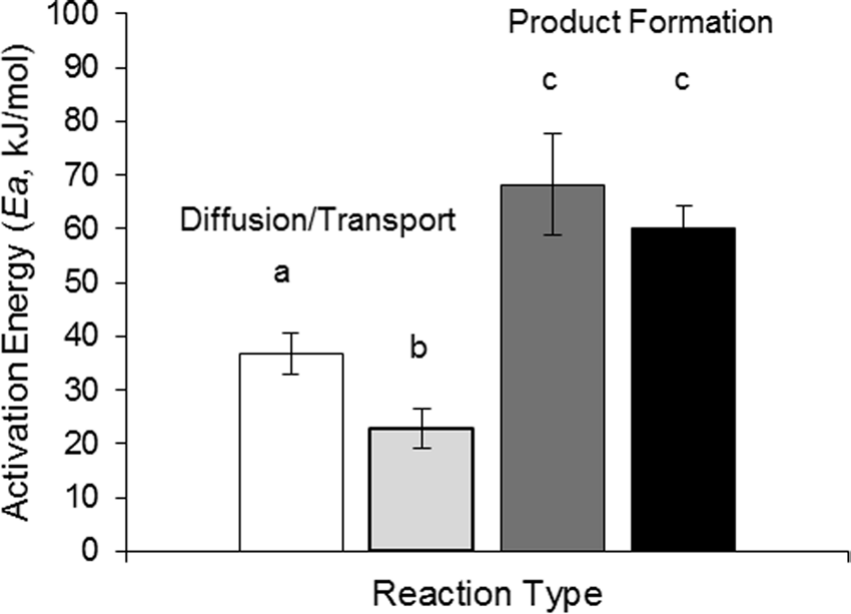
Activation energies, *E*_*a*_, (mean + s.e.m.) for different processes, diffusion and transport measured *in vitro* (white, *N* = 17) and *in vivo* (light gray, *N* = 23) and product formation *in vitro* (dark gray, *N* = 34) and *in vivo* (black, *N* = 18). Differences in lowercase letters above each bar indicate significant (*P* < 0.05) contrasts between means following ANOVA.

In a meta-analysis of Arrhenius temperature relationships, I compared activation energies (kJ/mol), *E*_*a*_, for diffusion or transport processes versus product formation in enzyme-catalyzed reactions (see Methods). ANCOVA analysis (see Supplementary Information, Table S1) revealed that process type (diffusion/transport versus product formation) (*F*_1,109_ = 15.74, P < 0.001) and, for diffusion and transport processes, *in vivo* versus *in vitro* environments (*F*_3,109_ = 3.2, *P* = 0.01) influenced *E*_*a*_. These outcomes suggest that diffusion or transport is less temperature-sensitive than product formation, as indicated by a mean *E*_*a*_ one half of that of product formation (Fig. 1). Interestingly, these results suggest that the difference in temperature sensitivity between process types is greater in more crowded *in vivo* environments, as would be expected if greater friction from molecular collisions reduce the temperature sensitivity of diffusion and transport^6,19^.

### Temperature-dependent reaction-diffusion model

Following Equations (1–3) and the difference in estimated *E*_*D*_ and *E*_*Z*_ (Fig. 1), it can be hypothesized that, as temperature increases, limits to the diffusion or transport of products (and for exothermic reactions, heat) may lead to accumulation of products and decrease reaction rate. To understand such possible consequences, it is necessary to explicitly add diffusion or transport to the reaction description. While previous studies show how arbitrary product concentrations influence the enzyme kinetic parameters that maximize reaction rate^22,23,30^, adding diffusion and transport explicitly allows the difference in *E*_*D*_ and *E*_*Z*_ to freely determine substrate and product concentrations and thus the rate of reaction at steady-state.

**Figure 2:**
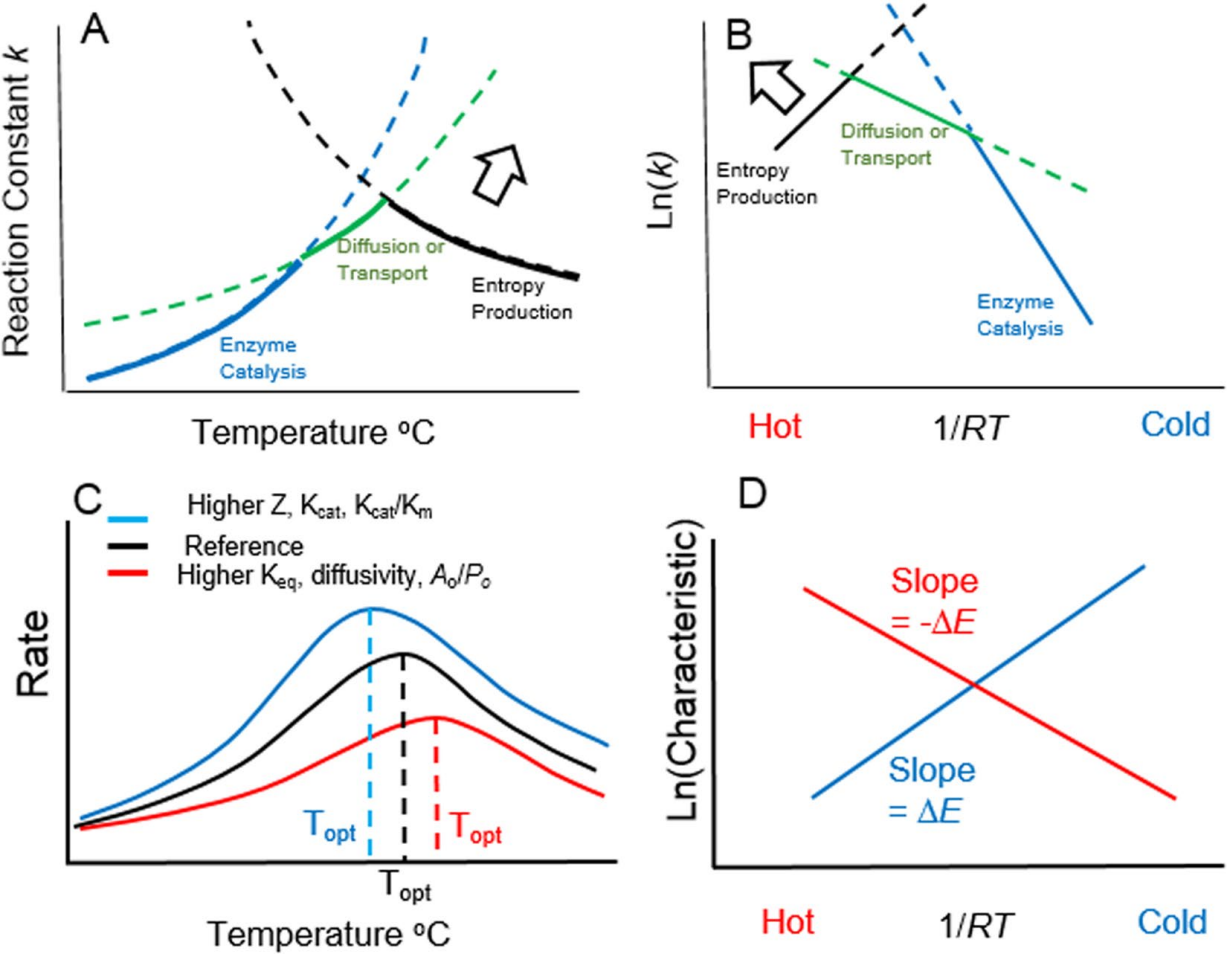
General qualitative predictions of the shifting factor model for reaction constant (*k*) versus temperature (**A**,**B**, Equations 2 and 3) and the resulting reaction-diffusion thermodynamic model of steady-state biochemical reaction rate (**C**,**D**, Equations 11 and 13). (**A**) Reaction rate (*k*) determined by shifts in limiting factors from enzyme catalyzed product formation (blue), to diffusion or transport of substrate (green) to entropy production (black) with increasing temperature. Solid curves indicate temperatures where that factor is limiting, dashed curves where it is not. (**B**) Relationships in A. expressed as Arrhenius functions (ln(*k*) versus 1/*RT*). Broad arrows in (**A**) and (**B)** indicate the change in the entropy-production-limited rate that results if entropy production is increased. (**C**) Smoothed qualitative relationships of reaction constant versus temperature, as influenced by reaction characteristics, yielding shifts in optimal temperatures, *T*_*opt*_: reference condition (black), decreasing *T*_*opt*_ in response to (blue) higher enzyme concentration, *Z*, catalytic capacity, *K*_*cat*_, and efficiency, *K*_*cat*_*/K*_*m*_; increasing *T*_*opt*_ in response to (red) higher reaction favorability, *K*_eq_, diffusivity, *d*_0_, and ratio of substrate *A*_*o*_ to product *P*_*o*_ outside the reaction site. (**B**) Expected general form of Arrhenius relationships for ln (characteristic) versus 1/*RT* (T in °K) for cooler *T*_*opt*_ associated with higher *Z*, *K*_*cat*_, and *K*_*cat*_*/K*_*m*_ (positive slope = Δ*E*, blue line) and hotter *T*_*opt*_ associated with higher *K*_*eq*_, d_0_, and *A*_*o*_/*P*_*o*_ (negative slope = −Δ*E*; red line).

The inclusion of diffusion or transport, and the difference in temperature sensitivity with product formation, leads to a hypothetical shift in the factor limiting the reaction rate from catalysis by enzymes (*k*, Equation (3)) to diffusion or transport (D, Equation (3)), to entropy production (Equation (2)) as temperature increases (Fig. 2A). Thus the overall temperature response of reaction rate may represent the shifting imposition of these limiting factors on the reaction coefficient. Plotting reaction rates as linear Arrhenius relationships shows this shift more readily as the change in slopes associated with the shift in limiting factors (Fig. 2B). Interestingly, this pattern is consistent with the marked reduction in or reversal of the sign in slope of many Arrhenius relationships of enzyme activity as temperatures approach and exceed *T*_*opt*_.^2,8,31^. For noisy experimental data, the overall relationship may appear as a smooth curve (Fig. 2C). The shift to limitation by entropy production can occur even in the absence of diffusion limitation of the reaction constant, as activity a* reflected in product to substrate ratios can still be temperature dependent. Thus, the derivations that follow are insensitive to whether diffusion coefficient or reaction constant are used (see Methods).

Under this limiting factor transition model, at higher temperatures, it is possible that reactions may be limited by entropy production to the surroundings and decline at temperatures below *T*_*den*_. To evaluate, I consider a simplified reaction-diffusion system appropriate for an exothermic single or coupled reaction^32^, that is, an overall *K*_*eq*_ > 0. Previous theoretical work suggests two types of reaction-diffusion systems for study: (1) reversible reactions described as a series of first-order conversions among substrate, one or more transition states, and products, or (2) reactions that convert substrate into product without explicit consideration of intermediates, and instead use second- or higher-order processes, sometimes derived from considering reversible steps^21,26,31^. Here I choose the latter approach because entropy changes are well-known to be “path independent” and driven by the difference in potential from substrate to product, regardless of intermediate steps (different paths). Furthermore, reaction reversibility is included in both the calculation of chemical activity^2,20,24^ and in second-order processes that incorporate reverse reactions that I will consider here, such as Michaelis-Menten dynamics^24,33^.

Suppose a substrate of concentration *A*_o_ outside the reaction site diffuses or is transported to a reaction site (location *i* to represent inside the site) with substrate concentration *A*_*i*_. At the site, substrate conversion to product, with concentration *P*_*i*_, is catalyzed by an enzyme at concentration Z. Product is then diffused or transported outside the reaction site relative to a surrounding product concentration *P*_*o*_. According to a simple reaction-diffusion description of this process:

4 $$\begin{array}{c}{\rm{d}}{A}_{i}/{\rm{d}}t=D({A}_{o}\mbox{-}{A}_{i})\mbox{-}f(k,{A}_{i},Z)\\ {\rm{d}}{P}_{i}/{\rm{d}}t=f(k,{A}_{i},Z)\mbox{-}D({P}_{i}\mbox{-}{P}_{o}),\end{array}$$

where *D* is a diffusion coefficient and *k* is a reaction constant. The function *f* is the rate of product formation and can be a first-order reaction or a second-order process (such as Michaelis-Menten) that includes mass action of substrate and enzyme and reaction constant with at least one reversible step (enzyme-substrate complex back to substrate).

At steady-state (where d*A*_*i*_/d*t* and d*Pi*/d*t* equal zero), a* is the ratio of product concentration *P*_i_* to substrate concentration *A*_i_* divided by *K*_*eq*_, the ratio of product to substrate at equilibrium, or when the forward reaction equals the reverse reaction.

5 $${a}^{\ast }={P}_{i}^{\ast }/({A}_{i}^{\ast }{K}_{eq})$$

I assume the reaction system (reaction site and surroundings) are isothermal, that is, the temperature of the surroundings is not changed by the heat of the reaction. This would be analogous to live cells^20,25^ or membranes on organelles in liquid^34^. Total entropy production^25,29^ for the reaction-diffusion system is

6 $${{\rm{\sigma }}}_{{\rm{tot}}}^{\ast }={{\rm{\sigma }}}_{{\rm{D}}}^{\ast }+{{\rm{\sigma }}}_{{\rm{Z}}}^{\ast }$$

where σ*_D_ is entropy production due to diffusion or transport of both substrate and products^25,29,35^, and σ*_Z_ is entropy production from heat dissipation and product formation^24,25,36^. For σ*_Z_, Equation (2) can be rearranged to express total entropy production as a function of free energy, reaction constant, and temperature at steady state, and the reaction constant, k* can depend on either diffusion or transport or kinetics.

7 $$\begin{array}{ccc}{\sigma }_{{\rm{D}}}^{\ast } & = & RD{({A}_{o}{{-}}{A}_{i}^{\ast })}^{2}+RD{({P}_{i}^{\ast }-{P}_{o})}^{2};\\ {\sigma }_{{\rm{Z}}}^{\ast } & = & -\,Q/T+{\sigma }_{{\rm{C}}}=-{\rm{\Delta }}G{k}^{\ast }/T=Rln(1/{a}^{\ast }){k}^{\ast },\end{array}$$

Generalized temperature-dependent steady-state concentrations for first- and second-order reactions (see Methods and Supplementary Information) are obtained from solving Equation (2) at steady-state, yielding a temperature-dependent activity and steady-state reaction constant (see Methods)

8 $$1/{\rm{a}}\cong {{\rm{e}}}^{{\rm{\Delta }}{\rm{E}}/{\rm{RT}}}[{\rm{\Omega }}({d}_{0},{k}_{0},{A}_{o},Z){K}_{eq}]/{\rm{\Theta }}({P}_{o},{A}_{o},{k}_{o},{d}_{o},Z)$$

9 $${k}^{\ast }={d}_{0}{e}^{-{E}_{D}/RT}$$

Equation (8) shows that the entropy-production-limited reaction constant *k*_*S*_ will decrease with increasing temperature because substituting the steady-state activity into Equation (2) yields *k*_*S*_ ∝ e^−ΔE/RT^. Equation (9) shows that, as hypothesized, substrate concentrations and reaction rate at steady-state may become limited by diffusion or transport at higher temperatures rather than by enzyme-substrate binding and product formation, (Fig. 2A,B). The operative reaction coefficient at steady-state is effectively the diffusion coefficient. *T*_*opt*_ will occur where the diffusion-limited reaction constant (k*, Equation (9)) equals the entropy production- limited reaction constant (*k*_*S*_, Equation (2)) (Fig. 2A,B). Increasing entropy production effectively moves the function for *k*_*S*_ farther from the origin, allowing both *k*_*S*_ and *T*_*opt*_ to increase.

Substituting Equation (5) for a*, Equation (9) for k* in Equation (7), and Equations (13) and (15) from Methods for *A*_i_* and *P*_i_* in Equation (5) and simplifying (see Supplementary Information) yields expressions for each entropy source in Equation (6). Executing the sum yields the approximate net rate of change in total entropy produced in the surroundings of the reaction site:

10 $${{\rm{\sigma }}}_{{\rm{T}}}^{\ast }=R{d}_{0}{e}^{-{E}_{D}/RT}[{\rm{\Delta }}E/RT+\,\mathrm{ln}({\rm{\Omega }}{K}_{eq}/{\rm{\Theta }})+{A}_{o}],$$

where Ω and ϴ are the functions described in Equation (8) where Ω/ϴ decreases with greater external product concentration *P*_*o*_, enzyme catalytic capacity *k*_0_ and concentration *Z*, and increases with greater diffusivity *d*_0_ and external substrate concentration *A*_*o*_.

Equation (2) suggests that reaction rate may be maximized by maximizing the rate of entropy production in the surroundings, as suggested by other theoretical studies^21,24,25^. Maximizing steady-state σ*_T_ with respect to temperature, *T*, (∂σ*_T_/∂*T* = 0) yields an optimal temperature.

11 $${T}_{opt}=\frac{{\rm{\Delta }}E}{R(\frac{{\rm{\Delta }}E}{{E}_{D}}-\,\mathrm{ln}(\frac{{\rm{\Omega }}}{\Theta }{K}_{eq})-{A}_{o})}$$

Equation (11) states the reaction-diffusion thermodynamics, or RDT, hypothesis for temperature and predicts that *T*_*opt*_ will depend on cell environmental conditions (*A*_*o*_, *P*_*o*_, *Z, d*_*o*_) and specific reaction or enzyme characteristics (*K*_*eq*_, *K*_*cat*_, *K*_*m*_) (Fig. 2). None of these predictions are made explicitly by molecular state change mechanisms.

Alternatively, reaction characteristics and enzymes might maximize free energy/time (power), under the idea that greater biochemical work yields greater growth and reproduction. Power, *J*, is

12 $$J={{\rm{\sigma }}}_{{\rm{INT}}}^{\ast }T=R{A}_{o}{d}_{0}{e}^{-{E}_{D}/RT}({\rm{\Delta }}E/R+T[\mathrm{ln}({\rm{\Omega }}{K}_{eq}/{\rm{\Theta }})+{A}_{o}]).$$

However it is easily shown that there is no optimum power with respect to *T*, as power continually increases with *T*.

The RDT hypothesis manifests in two ways: (1) reaction rate as a unimodal function of temperature (Fig. 2A), and whose optimum shifts with enzyme characteristics and environmental substrate and product concentrations, and (2) as a series of predicted linear Arrhenius relationships (Fig. 2B) between reaction characteristics and 1/*RT*_*opt*_. For example, *T*_*opt*_ should increase for more thermodynamically favorable reactions (higher *K*_*eq*_). Solving Equation (11) for ln(*K*_*eq*_) yields an Arrhenius function (Equation 13) with slope −Δ*E* and intercept Δ*E/E*_*D*_−ln(Ω*K*_*eq*_*/*ϴ)−2*A*_*o*_

13 $$\mathrm{ln}({K}_{eq})=-\,{\rm{\Delta }}E(1/R{T}_{opt})+[{\rm{\Delta }}E/{E}_{D}\mbox{-}\,\mathrm{ln}({\rm{\Omega }}/{\rm{\Theta }})\mbox{-}{A}_{o}].$$

**Figure 3:**
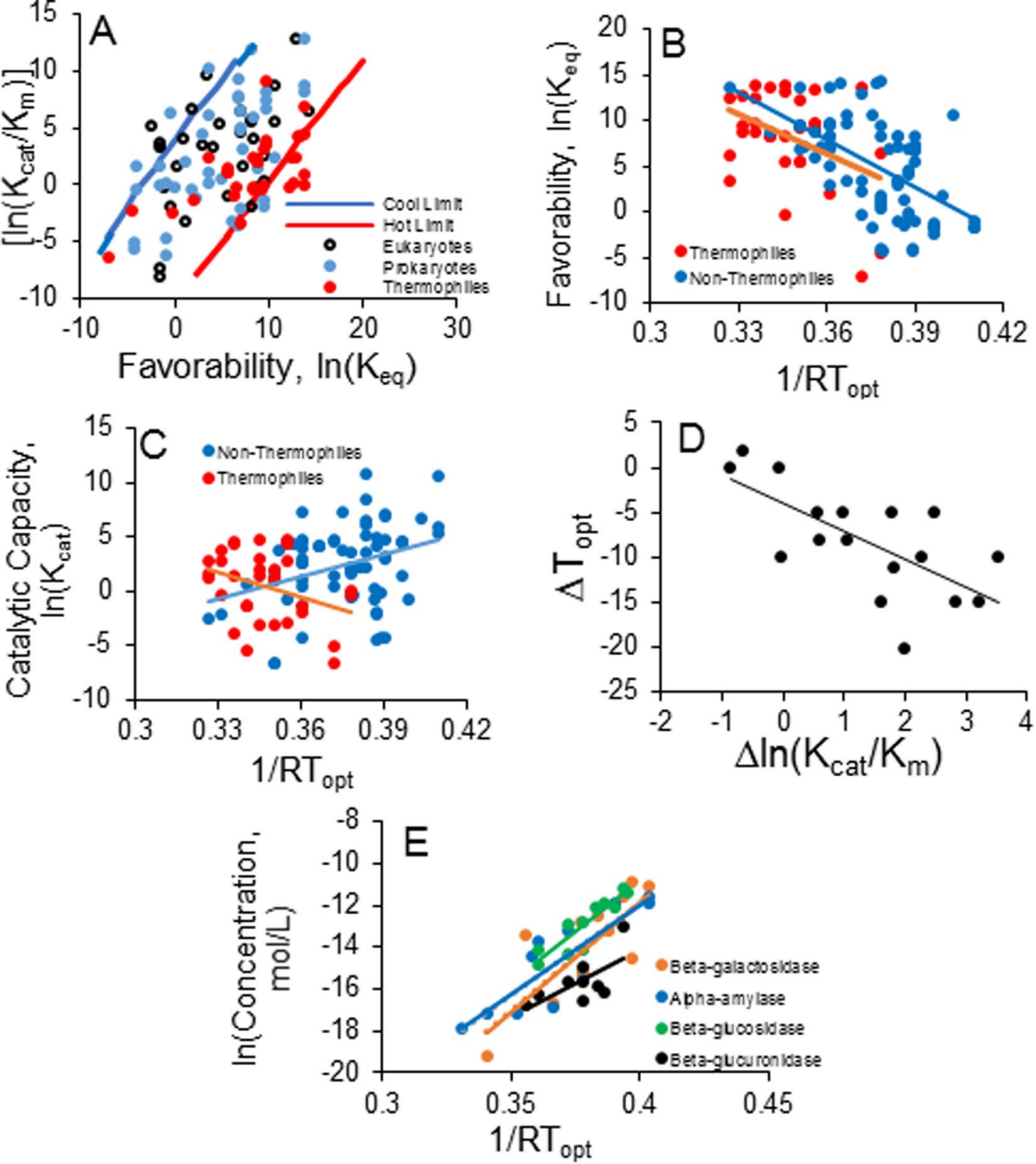
Meta-analysis of data testing the reaction-diffusion thermodynamic (RDT) hypothesis, based on qualitative predictions derived from Equations (11) and (13) (Fig. 2D). (**A**) Theoretical limits to reaction favorability, ln(*K*_*eq*_), and enzyme efficiency ln(*K*_*cat*_/*K*_*m*_) to achieve *T*_*opt*_ ranging from 0 °C (Cold Limit) to 100 °C (Hot Limit), under reported diffusivity and enzyme concentrations^1,2,37^. Data points are for enzymes from mesophile (blue, *N* = 54) and thermophile (red, *N* = 28) Prokaryotes and from Eukaryotes (open circles, *N* = 31). (**B**) Arrhenius regressions (see Supplementary Information for equations) of reaction favorability, ln (*K*_*eq*_) versus 1/*RT*_*opt*_ (°K) for Prokaryote and Eukaryote (combined) non-thermophile enzymes, blue, *N* = 85, *R*^2^ = 0.31, *P* < 0.001) and thermophile enzymes (red, *N* = 28, *R*^2^ = 0.16*, P* = 0.032). (**C**) Regressions of ln (*K*_*cat*_), for combined non-thermophile (blue, *N* = 78, *R*^2^ = 0.11, *P* = 0.005) and thermophile enzymes (red, *N* = 28, *R*^2^ = 0.13, *P* = 0.06). (**D**) Regression of change in *T*_*opt*_, Δ*T*_*opt*_ (°C) induced by experimental change in enzyme efficiency (Δ*K*_*cat*_/*K*_*m*_) (*N* = 17, *R*^2^ = 0.46, *P* = 0.003). (**E**) Significant (all *P* < 0.02) Arrhenius regressions of enzyme concentration, ln(*Z*), versus 1/*RT*_*opt*_(°K)) for each of four different hydrolytic enzymes: β-galactosidase (orange circles, *N* = 13); α-amylase (blue circles, *N* = 11), β-glucosidase (open squares, *N* = 11); β-glucuronidase (open circles, *N* = 9).

A similar Arrhenius form with a negative slope (implying higher T_opt_ at higher model parameter values) is obtained for diffusivity, ln(*d*_0_) and higher ratio of external substrate to product, ln(*A*_*o*_/*P*_*o*_) (Fig. 2B). In contrast, functions for enzyme concentration, ln(*Z*), and temperature-normalized reaction constant, ln(*k*_0_), have a positive slope, Δ*E*, implying that higher parameter values correspond to a lower *T*_*opt*_. In general, *T*_*opt*_ is negatively related to factors that increase the rate of product formation and positively related to factors that increase the concentration gradient (*A*_*o*_/*P*_*o*_) driving the forward reaction as well as reaction favorability and greater diffusivity. The RDT hypothesis also predicts a restricted subset of possible *k*_0_, *K*_*eq*_, and *Z* (Fig. 3A) that yield biologically realistic 0 < *T*_*opt*_* < *100 °C (see Methods for details in parameterizing the model).

The predictions of the RDT hypothesis were tested with a second meta-analysis of enzyme kinetic studies (*N* = 121) using enzymes from thermophile and non-thermophile Prokaryotes and Eukaryotes and including catabolic (hydrolysis, oxidation) and anabolic reactions (see Methods). 82% of observed *K*_*cat*_*/K*_*m*_ and *K*_*eq*_ combinations fell within the limits predicted by the RDT hypothesis (Fig. 3A), suggesting reaction-diffusion thermodynamics may constrain feasible enzyme efficiencies and types of reactions at different temperatures. Other factors can place constraints on enzyme properties, such as the need to avoid overly rapid or slow reactions in biochemical networks and cycles^30^, or to avoid product inhibition under environmental or cell physiological conditions of low product demand^22,23^. Here, the reaction-diffusion model allows product concentration to freely change and determine, for a given set of enzyme characteristics, what temperature would maximize reaction rate, and further, what enzyme characteristics maximize reaction rate at feasible biological temperatures, including cold and hot extrema. This suggests the hypothesis for further research that reaction-diffusion thermodynamics may place broad bounds on potential enzyme catalytic capacity and efficiency, while additional constraints may further narrow these possibilities.

In the meta-analysis, less favorable reactions exhibited lower *T*_*opt*_, as revealed by significant negative Arrhenius relationships between ln(*K*_*eq*_) versus 1/*RT*_*opt*_, (ANCOVA, *F*_1,106_ = 6.97, *P* = 0.002) for non-thermophiles and thermophiles combined (*P* < 0.03) (Fig. 3B) (see Supplementary Table S2). Anabolic or synthesis reactions had dramatically lower favorability (*F*_1,71_ = 47.1, *P* < 0.001) with mean ln(*K*_*eq*_) = −0.45 ± 0.78 s.e.m., N = 15), than hydrolysis reactions (Mean ln(*K*_*eq*_) = 7.0 ± 0.61 s.e.m., *N* = 56). As expected, anabolic or synthesis reactions exhibited significantly lower *T*_*opt*_ (°C) (Mean = 35.3 ± 2.9 s.e.m.) (*F*_1,71_ = 30.3, *P* < 0.001) than hydrolysis reactions (Mean = 53.8 ± 2.3 s.e.m.).

Other RDT model predictions were also substantially supported by relationships between reaction characteristics and *T*_*opt*_. As predicted, *T*_*opt*_ declined with greater enzyme catalytic capacity for non-thermophiles (Fig. 3C). A significant interaction occurred between thermal strategy and catalytic capacity, *K*_*cat*_, (*F*_2,96_ = 4.16, *P* < 0.001), as ln(*K*_*cat*_) decreased (*R*^2^ = 0.11, *P* = 0.005) with hotter *T*_*opt*_ for non-thermophiles, but increased for thermophiles (*R*^2^ = 0.16, *P* = 0.03) (Fig. 3C, Supplementary Table S3). Nevertheless, mean ln(*K*_*cat*_) for thermophiles (0.45 ± 0.98 s.e.m, *N* = 28) was significantly lower than that for non-thermophiles (4.2 ± 1.01 s.e.m, *N* = 85) (*t* = 2.07, df = 111, *P* = 0.043), indicating that thermophile enzymes that catalyze more favorable reactions with higher T_opt_ may have lower catalytic capacity. Thermophiles may also adjust reaction features other than enzyme efficiency, such as membrane permeability or lower enzyme concentrations^37^ to compensate for higher temperatures.

Further support for RDT predictions was provided by experimental reduction of enzyme efficiency (Fig. 3D) and concentration (Fig. 3E). As predicted the magnitude of decrease in enzyme efficiency from various manipulations was strongly related to accompanying increases in *T*_*opt*_ (*R*^2^ = 0.54, *P* = 0.003) (Fig. 3D). Across studies, greater enzyme concentrations for four different enzymes were strongly associated (*R*^2^ > 0.65, *N* > 9, *P* < 0.04) with lower *T*_*opt*_ (Fig. 2E) (for statistical details see Supplementary Information).

The outcomes of the reaction-diffusion model presented are preliminary, in the sense that the model does not explicitly include often-important details of reactions, such as the influence of product concentration on the reverse reaction or the effect of transitions of substrate to product among different enzyme complexes^4,8,22^. Such realities are beyond the scope of the general assessment presented here and would likely require simulation and be more meaningful if used to explore specific single reactions or reaction networks^22,27^. Nevertheless, the patterns observed in the meta-analysis (Fig. 3) strongly suggest that simplistic mechanisms may capture the essential processes that drive the influence of temperature on reactions.

The RDT hypothesis and supporting data contrast clearly with patterns expected from molecular state change mechanisms, which are largely independent of reaction characteristics^4,38^. Progressively steeper declines in enzyme activity over time with temperature above a threshold *T* are typically used to infer the threshold as *T*_*den*_^1,11,14^, as loss of enzyme function is assumed to explain the decline in activity. The RDT hypothesis instead suggests that more rapid declines in activity as *T* increases may be due to product inhibition^4^ even in very short-term measurements, and explains why *T*_*opt*_ might increase with immobilization (Fig. 2D), which often decreases enzyme efficiency. Likewise decreases in *T*_*opt*_ with enhancement^5,10,14,15^ or increased concentration (Fig. 2E) of the same enzyme are not easily explained by molecular state change mechanisms.

Molecular state changes, regardless of mechanism, might still explain declines in biochemical reaction rates at high temperatures in instances where diffusion and/or entropy production are not limiting. Such conditions might be true for low concentrations of enzymes catalyzing highly favorable reactions, such as hydrolysis, or in experimental situations where there are few limits to diffusion. For example, responses of microbial respiration to temperature appear well-explained by models including only the loss of heat capacity between enzyme-substrate and transition states, reflecting a large increase in internal entropy at higher temperatures^31,38,39^. This shift in limiting mechanism may extend to plants, where respiration appears to have much higher *T*_*opt*_ (41–50 °C)^17^ than photosynthesis, a series of coupled synthesis reactions^40,41^ optimized at temperatures < 30 °C. The *T*_*opt*_ range for photosynthesis is well below the *T*_*opt*_ expected from measured changes in heat capacity with increasing *T* of RuBisCo^42^, the enzyme catalyzing the most frequently rate-limiting carboxylation step in photosynthesis. The *T*_*opt*_ range for plant respiration, however, corresponds roughly to that expected from changes in heat capacity between enzyme-substrate transition states^43^. Another example is provided by the hydrolysis-driven reactions in the mitochondria of endotherm eukaryotic cells that maintain local mitochondrial temperatures near 50 °C in contrast to temperatures 35–37 °C in surrounding cytoplasm where mainly protein synthesis occurs^44^.

Macromolecular state changes and RDT mechanisms are not necessarily mutually exclusive, as they may reduce reaction rate at different temperatures, and the mechanism reducing reaction rate at the lowest temperature may best explain observed data. Consequently it is possible that respiration, dominated by highly favorable reactions, may be limited by macromolecular state changes, while less favorable synthesis reactions might be driven by reaction-diffusion thermodynamics^31,43^. Combining mechanisms of entropy production to surroundings with mechanisms of internal entropy increases, such as changes in charge distribution or heat capacity associated with the catalytic process^31^ would seem a fruitful area of future research.

The new, dynamic *T*_*opt*_ predicted by reaction-diffusion thermodynamics and reaction characteristics (Equation 11), has widespread implications for the physics of living systems^1,2,20,26,45^, for the origin of life^20^, and for boundaries on the efficiency and structure of evolved catalytic enzymes^31^. There are likely many potential industrial, environmental, and medical applications, such as optimizing yields of genetically modified organisms that overexpress catalytic enzymes^46^, understanding the role of fever in combating pathogens^47^, and elucidating potential consequences of climate change, such as why organisms exhibit weaker temperature sensitivity at temperatures 25–50 °C^11,45^ or why crops subject to warming and elevated CO_2_ have lower nutritional quality to humans^48^. A deeper understanding of biological temperature dependence may benefit from greater consideration of thermodynamic limits to cellular processes beyond the enzyme catalytic process, such as diffusion and transport of reaction substrates and products as well as heat dissipation^2,20,25^.

## Methods

### Meta-analysis of activation energies

Web of Science^®^ was searched for the keyword string (activation and energy and temperature and biochem* and react*), which yielded 115 results. Only 78 of these studies, with 92 independent estimates of *E*_*a*_, met criteria for inclusion: reported at least four measurements of rate and temperature and had *R*^2^ > 0.90 for the regression of ln(rate) versus 1/*RT*. In some studies, regression was performed by the author. Activation energies, *E*_*a*_, were used as reported for temperatures <30 °C to avoid issues associated with changes in slope of Arrhenius relationships at higher temperature. Estimates were classified as measured in living organisms (*in vivo*) (*N* = 41) or in containers (*in vitro*) (*N* = 51) and whether they applied to a diffusion or transport process (*N* = 40) or an enzyme catalyzed reaction (*N* = 52). Reactions included oxidation, hydrolysis, reduction and synthesis for both *in vivo* and *in vitro* experiments.

### Meta-analysis of *T*_*opt*_ and reaction characteristics

The MEP predicted Arrhenius relationship between optimal 1/*RT*_*opt*_ and reaction favorability, ln(*K*_*eq*_), and enzyme efficiency, ln(*K*_*cat*_/*K*_*m*_) was explored with a Web of Science^®^ search using the keyword string (enzyme* and optim* and temperature and biochem* and (“turnover number” or K-cat or K-eq or “equilibrium constant”)). This search yielded 338 studies of *T*_*opt*_ accompanying measurements of reaction kinetic parameters. Few studies measured or reported *K*_*eq*_, so these values were obtained for the substrate in each study from the NIST thermodynamics of enzyme catalyzed reactions database, https://randr.nist.gov/enzyme/. Further restriction to include only papers published since 2013 (to standardize methods for isolating proteins) and those that determined both *K*_*cat*_, the turnover number, and *K*_*m*_, the half-saturation constant, in the same study yielded 121 studies featuring 175 separate experiments. The final set included enzymes from eukaryotes (*N* = 32), mesophilic prokaryotes (*N* = 65), and thermophilic prokaryotes (*N* = 28). Psychrophilic (cold-adapted) prokaryotes accounted for only 9 of the original 338 studies, and so were not analyzed due to small sample size. Seventeen of the 125 studies compared *T*_*opt*_ for enzymes that differed in their efficiency due to immobilization or chemical enhancement. These studies allowed the difference in *T*_*opt*_ to be related to difference in enzyme efficiency (*K*_*cat*_/*K*_*m*_, in s^−1^mM^−1^). In all studies, measurements of *K*_*cat*_ and *K*_*m*_ were standardized to 25 °C to allow comparison between studies. This temperature correction multiplied the reported *K*_*cat*_ and *K*_*m*_ at their measured temperature, *T*_*m*_ (°K) by the function exp(-(*E*_*a*_*/R*)[(1/298) – (1/*T*_*m*_)]), where *E*_*a*_ equaled the average *in vitro* activation energy (kJ/mol) from the meta-analysis (Fig. 1) (71 kJ/mol), *R* is the gas constant 8.32 × 10^–3^ kJ.mol^−1^K^−1^ and 298 is 25 °C in °K.

A separate Web of Science^®^ search was conducted to test for the effect of changing enzyme concentration on *T*_*opt*_. Keywords (enzyme* and concentration and optim* and temperature), yielded 915 results. These were further searched for sets of at least six studies of *T*_*opt*_ each for different, particular enzyme-substrate pairs in order to show the relationship between enzyme concentration and 1/*RT*_*opt*_, standardized for other factors. Only four hydrolytic enzyme-substrate pairs met these criteria: beta-galactosidase with ortho-nitrophenyl beta-galactoside, beta-glucosidase with p-nitrophenyl-D-pyranoglucoside, beta-glucuronidase with 4-nitrophenyl-β-d-glucuronide, and alpha-amylase with starch. Arrhenius relationships between ln(enzyme concentration) and 1/*RT*_*opt*_ were determined for each enzyme, and the overall relationship for all four combined was analyzed with ANCOVA (see Statistics and Supplementary Information).

### Limits to feasible reaction characteristics

Equation (11) was parameterized to estimate combinations of K_eq_ and *k*_*o*_ (empirically *K*_*cat*_/*K*_*m*_), not standardized for temperature but instead measured at *T*_*opt*_, that would yield feasible 0 < *T*_*opt*_ < 100 °C. Enzyme concentrations, *Z* (mol/L), were between 10^−9^–10^−7^, as reported in studies from the meta-analysis. Diffusivity (cm^2^/s), *d*_0_, in aqueous solutions^1,18^ ranges between 10^−6^ and 10^−5^. Outside substrate concentration, *A*_*o*_ ranged between 10^−5^ and 10^−3^ mol/L. Outside product concentration, *P*_o_, was assumed to be 10% of *A*_o_. Mean activation energy (kJ/mol) for *in vitro* activation energy for diffusion/transport from the meta-analysis (Fig. 1) was used for *E*_*D*_. The difference between estimated *E*_*D*_ and *E*_*Z*_ (activation energy for product formation), also estimated from the meta-analysis for *in vitro* reactions, was used as Δ*E*. *R* is the gas constant, 8.318 × 10^−3^ kJ mol^−1^K^−1^. Estimates of *T*_*opt*_ were made for varying values of *K*_*eq*_ and *K*_*cat*_/*K*_*m*_ that produced *T*_*opt*_≅0 °C (Cold Limit) under the high extreme for enzyme concentration and low extreme for diffusivity, and *T*_*opt*_≅100 °C (Hot Limit) under the low extreme for enzyme concentration and the high extreme for diffusivity (Fig. 3A).

### Statistics

Mean activation energies for each classification were compared with One-way ANOVA followed by Tukey’s multiple comparison test for contrasts in SPSS 24 on ln-transformed data to avoid the influence of unequal variances among classes. ANCOVA was used to analyze Arrhenius relationships for variables ln(*K*_*eq*_), ln(*K*_*cat*_), and ln(*K*_*cat*_*/K*_*m*_) with covariate 1/*RT*_*opt*_, and Eukaryotes versus Prokaryotes or meso- versus thermophiles as fixed factors. The relationship between ln(enzyme concentration, mol/L) and ln(*A*_*o*_) (substrate concentration) versus 1/*RT*_*opt*_ for the four catalytic enzymes was also analyzed with ANCOVA, with enzyme as a fixed factor, 1/*RT*_*opt*_ as a covariate and an interaction term. Individual linear regressions were also conducted between ln(enzyme concentration, mol/L), substrate concentrations and 1/*RT*_*opt*_. All comparisons were two-tailed, with α = 0.05.

### Model of Reaction-Diffusion Temperature Dependence

An enzyme at concentration Z, can catalyze rates of change in concentrations of substrate *A*_*i*_ and product *P*_*i*_ at the reaction site *i* (Equation (4) in Results.

$$\begin{array}{c}{\rm{d}}{A}_{i}/{\rm{d}}t={D}_{A}({A}_{o}-{A}_{i})-f(k,{A}_{i},Z)\\ {\rm{d}}{P}_{i}/{\rm{d}}t=f(k,{A}_{i},Z)-{D}_{P}({P}_{i}-{P}_{o})\end{array}$$

where _*D*i_ are the diffusion coefficients for substrate or product in or out of the reaction site, while *A*_*o*_, *A*_*i*_ and *P*_*o*_, *P*_*i*_ are substrate and product concentrations outside and at the reaction site, respectively, and *k* is a reaction constant. The function *f* is the rate of product formation, which increases with greater *k*, *A*_*i*_, and *Z*.

At steady state

14 $${A}_{i}^{\ast }=s({D}_{i},{A}_{o},k,Z);\,{P}_{i}^{\ast }={P}_{o}+g({D}_{i},{A}_{o},k,Z,{P}_{o})$$

in which *s*(*D*_*i*_, *A*_*o*_, *k, Z*) is a decreasing function of *k* and *Z*, but increasing function of *A*_*o*_ and *D*, and *p*(*A*_*o*_) is an increasing function of *A*_*o*_, *k, Z*, and decreasing function of *D*. After substituting Equations (3) as Boltzmann temperature dependent functions for *D*_*i*_ and *k* in the function *s*, it can be shown (see Supplementary Information) for first- and second-order reactions^49^, the, the ratio of the general functions *s*(*D*_*i*_, *A*_*o*_, *k, Z*) and *g*(*A*_*o*_*D*_*i*_, *A*_*o*_, *k, Z, P*_*o*_) is proportional to a Boltzmann exponential function, respectively:

15 $${P}_{i}^{\ast }/{A}_{i}^{\ast }=[\Theta ({A}_{o},Z,{k}_{o},{d}_{o},{P}_{o})]{e}^{-{\Delta }E/RT}/{\rm{\Omega }}({d}_{0},{k}_{0},{A}_{0},Z)$$

where ∆*E* = *E*_*Z*_ − *E*_*D*_≅30 kJ/mol (Fig. 1) and Ω increases with *d*_0_ and *A*_*o*_ and decreases with *k*_0_ and *Z* and ϴ is an increasing function of *A*_*o*_, *P*_*o*_*, k*_*o*_, and Z and decreasing function of *d*_*o*_. Consequently, a major novel result is that activity is strongly temperature-dependent and approximately proportional to a Boltzmann exponential function for first and second-order reactions (see Supplementary Information):

16 $${{\rm{a}}}^{\ast }\cong {\rm{\Theta }}{e}^{-{\rm{\Delta }}E/RT}/{\rm{\Omega }}{K}_{eq}]$$

The steady-state reaction rate r* is defined by

17 $${D}_{{\rm{A}}}({A}_{o}-{A}_{i}^{\ast })=f(k,{A}_{i}^{\ast },Z)$$

Substituting the function Equation (14) for *A*_i_* for different types of reaction descriptions (see Supplementary Information) and simplifying yields

18 $${{\rm{r}}}^{\ast }={d}_{0}{e}^{-{E}_{D}/RT}{A}_{o}$$

Note that r* becomes equivalent to k*A_*o*_ where k* = *d*_0_*e*^*−ED*/*RT*^, supporting the general assertions outlined in Equations (1), (2) and (6).

### Data availability

Additional mathematical and statistical analyses are available as Supplementary Information. Two database files, one entitled Ritchie Activation Energies.xls and the other Ritchie Enzyme Kinetics.xls, provide the reviewed data used in the two meta-analyses.

## Electronic supplementary material

Supplemental Information
