# Supplementary material for: Reaction and diffusion thermodynamics explain optimal temperatures of biochemical reactions
Source: Sci Rep. 2018 Jul 23;8:11105. doi: 10.1038/s41598-018-28833-9 (PMC6056565; doi:10.1038/s41598-018-28833-9)
Supplement: Supplementary file 1 — Supplemental Information [file 41598_2018_28833_MOESM1_ESM.docx]

**Reaction and diffusion thermodynamics explain optimal temperatures of biochemical reactions**

**By Mark E. Ritchie**

**Supplementary Information**

**Derivation of temperature-dependent chemical activity of simple reaction-diffusion systems.**

**Generalized reaction-diffusion system**. For a reaction catalyzed by an enzyme at concentration *Z*, subject to diffusion of substrate into a and product away from a reaction site, let changes in substrate *A_i_* and product *P_i_* concentrations at the reaction site be

d*A_i_*/d*t* = *D_A_*(*A_o_* – *A_i_*) – *f*(*k,A_i_,Z)*

d*P_i_*/d*t* = *f*(*k,A_i_,Z)* – *D_P_*(*P_i_* – *P_o_*) (S1),

where *D_j_*  are diffusion coefficients for substrate *A* or product *P,* respectively, *A_o_, A_i_* and *P_o_*, *P_i_* are substrate and product concentrations outside and inside the reaction site, respectively, and *k* is a reaction constant. The function *f* is rate of product formation, defined to be increasing with greater *k*, *A_i_,* and Z and can be first-order, Michaelis-Menten, or other form. At steady state

*A_i_** = *s*(*D_i_*,*A_o_*,*k*,*Z*)

*P_i_*=P_o_ + p(A_o_,D_i_*,*A_o_*,*k*,*Z*) (S2),

in which *s*(*D_i_,A_o_,k,Z*) is a decreasing function of *k* and *Z*, but increasing function of *A_o_* and *D,* and *p(A_o_)* is an increasing function of *A_o_*. For different forms of the function *f*, for example, we obtain:

**First-order reaction**

*f*(*k,A_i_,Z)* = *kA_i_*

*A_i_**= *DA_o_*/(*D* +*k*)

*P_i_* = P_o_* + *kA_o_/*(*D*+*k*)

*P_i_*/A_i_** = (*k*/*D*)(1+ *P_o_/A_o_*) +*P_o_/A_o_* (S3)

**Second order-order reaction, simple mass action**

*f*(*k,A_i_,Z)* = *kZA_i_*

*A_i_**= *DA_o_*/(*D* + *kZ*)

*P_i_* = P_o_* + *kZA_o_/*(*D* + *kZ*)

*P_i_*/A_i_** = *P_o_*/*A_o_*+(*kZ/D*)(*P_o_*/*A_o_*+ 1) (S4)

**Second-order reaction, Michaelis-Menten, no restrictions**

*f*(*k,A_i_,Z)* = *kZA_i_*/(*k_m_* + *A_i_*)

*A_i_** = *A_o_* – *k_m_* – *kZ*/*D*

*P** = *P_o_* + *kZA_i_*/*[(*k_m_*+*A_i_**)]

$\frac{{P_{i}}^{*}}{A_{i}^{*}}=\frac{1}{A_{o}-\frac{kZ}{D}}\left[ P_{o}+kZ+\frac{P_{o}k_{m}}{\left( A_{o}-\frac{kZ}{D} \right)-k_{m}} \right]$ (S5)

Note, the solution for *A_i_** is the solution to the quadratic equation:

(*A_o_* – *k_m_* – *kZ*/*D*)*A_i_* – *A_i_^2^* + *A_o_k_m_* (S6)

**First-order, explicitly reversible reaction**

*f*(*k,A_i_,P_i_*) = *k_f_A_i_ - k_r_P_i_*

where the function *f* is explicitly dependent on product concentration *P_i_* and the reaction constants *k_f_*, forward reaction, and *k_r_*, the reverse reaction

*A_i_** = [*A_o_*(*D* + *k_r_*) + *k_r_P_o_*]/(*D* + *k_f_* + *k_r_*)

*P_i_** = *DP_o_*/(*D* + *k_r_*) + *k_f_*[*A_o_*(*D* + *k_r_*) + *k_r_P_o_*]/(*D* + *k_f_* + *k_r_*)

$\frac{P_{i}^{*}}{A_{i}^{*}}=\frac{DP_{o}\left( D+k_{f}+k_{r} \right)}{\left( D+k_{r} \right)\left[ A_{o}\left( D+k_{r} \right)+P_{o}k_{r} \right]}+\frac{k_{f}}{D+k_{r}}$ (S7)

**Temperature dependence.** Assuming Boltzmann temperature dependence for *D_i_*, which is appropriate for diffusion in viscous fluids*^18,19,35^* and for *k* yields

*D = d_0_e^-ED/RT^* ; *k = k_0_e^-EZ/RT^* (S8),

where *d_0_* and *k_0_* are constants*^1^* roughly equivalent to temperature-corrected diffusivity and reaction turnover number (*K_cat_*), respectively. *R* is the gas constant, and *E_D_* and *E_Z_* are activation energies for diffusion/transport and product formation, respectively, where *∆E* = *E_Z_* – *E_D_*. The meta-analysis of activation energies (text, Fig. 1) shows clearly that Δ*E* > 0, averaging about 30 kJ/mol.

Equations (S8), inserted into the various solutions for *P*_i_* and *A_i_** in (S3-S5, S7) yield the following proportionalities that determine chemical activity *a** in the main text thermodynamic equations (7-12). In all cases, I assume that, for large *T* near *T_opt_* , 1+*h*(*T*) ∝ *h*(*T*), where *h*(*T*) is an exponential function of *T*. Therefore,

**First-order reaction (Equations (S3))**

*P_i_*/A_i_** = (*k*/*D*)(1+ *P_o_/A_o_*) +*P_o_/A_o_*

*P_i_*/A_i_** = (*k_o_*/*d_o_*)*e^-ΔE/RT^* (1+ *P_o_/A_o_*) +*P_o_/A_o_*

*P_i_*/A_i_** ∝ *e ^-ΔE/RT^* (S9)

**Second-order reaction, mass action, Equations (S4)**

*P_i_*/A_i_** = [*P_o_*+ (*kZ/D*)(*P_o_* + *A_o_*)]/*A_o_*

*P_i_*/A_i_** = [*P_o_*+ (*k_o_Z*/*d_o_*)*e^-ΔE/RT^* (*P_o_* + *A_o_*)]/*A_o_*

*P_i_*/A_i_** ∝ *e^-ΔE/RT^* (S10)

**Second-order reaction**, **Michaelis-Menten, Equations (S5)**

$\frac{{P_{i}}^{*}}{A_{i}^{*}}=\frac{1}{A_{o}-\frac{kZ}{D}}\left[ P_{o}+kZ+\frac{P_{o}k_{m}}{\left( A_{o}-\frac{kZ}{D} \right)-k_{m}} \right]$

$\frac{{P_{i}}^{*}}{A_{i}^{*}}=\frac{1}{A_{o}- \frac{k_{o}{Ze}^{-\Delta E/RT}}{d_{o}}}\left[ P_{o}+k_{o}{Ze}^{-E_{Z}/RT}+\frac{P_{o}k_{m}}{\left( A_{o}- \frac{k_{o}{Ze}^{-\Delta E/RT}}{d_{o}} \right)-k_{m}} \right]$ (S11)

Dividing numerator and denominator by the quantity α, where α = (*k_o_/d_o_*)*Z*e^-Δ^*^E/RT^*, yields approximately, where I assume *A_o_*/α >> 1 and (*A_o_*-*k_m_*) /α >> 1 (concentrations are larger than the kinetic constant) and *E_D_* ≅ Δ*E* (Fig. 1) so (*A_o_*/α) - 1 ≅ *A_o_*/α and ((*A_o_*-*k_m_*)/α) - 1 ≅ (*A_o_*-*k_m_*)*/*α

*P_i_**/*A_i_** ≅ (α/*A_o_*)[*P_o_* + *k_o_Z*e^-^*^EZ/RT^* + *P_o_k_m_*α/(*A_o_*-*k_m_*)]

≅ *P_o_*(α/*A_o_*) + ((*k_o_Z*)^2^/*d_o_A_o_*) e^-^*^ΔE/RT^* + *P_o_k_m_*α^2^/(*A_o_*(*A_o_*-*k_m_*)) (S12)

The non-α terms and exponentials implicit in (S12) are likely to be < 1 (since *A_o_* > *P_o_*), so the ratio *P_i_**/*A_i_** can be approximated by the lower order terms with exponents -Δ*E*/*RT*. Hence,

*P_i_**/*A_i_** ∝ *e^-ΔE/RT^* (S13)

**First-order, simple reversible reaction.** This reaction has much more complex behavior and interesting temperature sensitivity that depend on the magnitude of *k_f_, k_r_* and *D*. For the exothermic reactions emphasized in this paper, at high values of *T* near *T_opt_*, *D* ≅ *k_f_ >>k_r_*, Under these assumptions, Equation (S7) simplifies to

$\frac{P_{i}^{*}}{A_{i}^{*}}\cong\frac{P_{o}\left( D+k_{f} \right)}{\left[ A_{o}D \right]}+\frac{k_{f}}{D}$ (S14)

$\frac{P_{i}^{*}}{A_{i}^{*}}\cong\frac{P_{o}\left( {1+k}_{f,0}e^{-\Delta E/RT} \right)}{A_{o}}+\frac{k_{f,0}e^{-\Delta E/RT}}{d_{0}}$ (S15) which clearly shows that

*P_i_**/*A_i_** ∝ *e^-ΔE/RT^* (S16)

**Activity and Reaction Rate**

Consequently, the ratio *P_i_**/*A_i_** that defines is determined by the function for steady-state *A_i_**

*A*_i_*** = Ω(*d_0_,k_0_*,*A_0_*,*Z*)

*P_i_** = Θ(A_o_,*d_0_,k_0_*,*Z,P_o_*)*e ^-ΔE/RT^* (S17)

The function Ω increases with *d_0_* and *A_o_* and decreases with *k_0_* and *Z*, while the function ϴ where *∆E* = *E_Z_* – *E_D_* ≅ 30 kJ/mol (Fig. 1 in the main text). Consequently, the activity at steady-state is highly temperature-dependent:

*a** = Θ*e ^-ΔE/RT^ /* Ω*K_eq_* (S18)

Also, regardless of the reaction order and type for *f*(*k,A_i_*,Z)*, the reaction rate at steady-state is defined by

*r* = f*(*k,A_i_*,Z)* = *D*(*A_o_* – *A_i_**) (S19)

Substituting for *A_i_** yields, at higher temperatures (assumption of large *T*),

*r** ≅ *d_0_e^-ED/RT^A_o_* (S20)

**Temperature dependence of K_eq_**

Temperature affects Keq, which for exothermic reactions results from heat generated by the forward reaction increasing the rate of reverse reaction and thus reduces Keq. The magnitude of temperature influence on Keq is typically much smaller than the direct influence of temperature on the rate of molecular movement and molecular collision^1,50^ so I did not include it for simplicity. For exothermic reactions, Keq decreasing with increasing temperature would thus reduce the overall activity, *a**, for the same substrate and product concentrations. This would further amplify the temperature-inhibition of entropy production for exothermic reactions.

**Optimal temperature, *T**corresponds to a maximum entropy production**

Beginning with equation (9) from the text

σ*_tot_ = *RA_o_ d_0_ e^-ED/RT^* [Δ*E/RT* + ln(Ω*K_eq_*/ϴ) + 2*A_o_*] (S16)

Collecting constants by defining β = *A_o_*+ ln(Ω*K_eq_*/ϴ)]

σ*_tot_ = *RA_o_ d_0_* [β *e^-ED/RT^* + *e^-ED/RT^* Δ*E/RT*] (S17)

∂σ*_tot_/∂*T* = *A_o_ d_0_* [β *e^-ED/^*^RT^(*E_D_/RT*^2^) + *e^-ED/RT^*(Δ*E/RT*^2^) - *e^-ED/RT^*(Δ*EE_D_/R*^2^*T*^3^)]

Empirically (Fig. 1), Δ*E* ≅ *E_D_* so, taking the second derivative yields

∂σ*_tot_*^2^*/∂*T*^2^ = *A_o_ d_0_ e^-ED/RT^* [β (*E_D_*^2^*/RT*^3^) - 2β (*E_D_/RT*^3^) + 2(Δ*EE_D_/R*^2^*T*^3^) - (4Δ*E/T*^3^) - 3Δ*EE_D_*^2^*/R*^2^*T*^4^) + (4Δ*EE_D_/R*^2^*T*^4^)].

Simplifying

∂σ*_tot_*^2^*/∂*T*^2^ = (*A_o_d_o_ /RT^3^*) *e^-ED/RT^* [-2(α*E_D_* - Δ*E*) + (4 – α)Δ*EE_D_/RT* - Δ*EE_D_*^2^*/*R^2^T^2^)] (S18)

Because the ratios Δ*E/R* and *E_D_/R*  are an order of magnitude greater than *T*, given Fig. 1, the dominant term inside the bracket in Equation (S18) is -Δ*EE_D_*^2^*/*R^2^T^2^ which is negative, so the second derivative is negative and the optimum in Equation (11) is a maximum.

**Regressions for reaction characteristics**

Regressions for relationships in the meta-analysis of factors affecting optimal temperature in enzyme kinetic studies. All coefficients are shown with + s.e.m. in parentheses and *K_cat_*  and *K_m_* are temperature-corrected to 25^o^C.

1. *Reaction favorability (K_eq_)*

Non-thermophiles: ln(*K_eq_*) = -178.5(+ 72.1)(1/*RT_opt_*) +72.2(+ 28.6), *N* = 78, *R*^2^ = 0.33, *P*<0.001

Thermophiles: ln(*K_eq_*) = -209.65(+ 88.4)(1/*RT_opt_*) +81.0(+ 32.3), *N* = 28, *R*^2^ = 0.33, *P*=0.008

1. *Catalytic capacity (K_cat_)*

Non-thermophiles: ln(*K_cat_*) = 69.4(+ 32.8)(1/*RT_opt_*) – 23.7(+ 11.0), *N* = 78, *R*^2^ = 0.11, *P*=0.005

Thermophiles: ln(*K_cat_*) = -99.2(+ 39.1)(1/*RT_opt_*) +35.8(+ 12.3), *N* = 28, *R*^2^ = 0.16, *P*=0.032

1. *Enzyme efficiency* (*K_cat_*/*K_m_*)

Non-thermophiles ln(*K_cat_*/*K_m_*) = -11.6(+ 25.4)(1/*RT_opt_*) + 6.8(+ 21.3), *N* = 78, *R*^2^ = 0.001, *P*=0.92

Thermophiles: ln(*K_cat_*/*K_m_*) = -60.0(+ 35.3)(1/*RT_opt_*) +23.0(+ 14.7), *N* = 28, *R*^2^ = 0.07, *P*=0.201

1. *Experimental manipulation of enzyme efficiency*

Δ*T_opt_* = -3.1(+ 1.3)(Δ*K_cat_*/*K_m_*) - 4.0(+ 1.6), *N* = 17, *R*^2^ = 0.46, *P* = 0.003

1. *Enzyme concentration*

Beta-galactosidae: ln(*Z*) = 104.1(+ 25.8) (1/*RT_opt_*) – 53.5(+ 25.8), *N* = 13, *R^2^* = 0.60*, P* = 0.002

Alpha-amylase: ln(*Z*) = 86.3 (+ 16.6) (1/*RT_opt_*) - 46.5 (+ 6.1), *N* = 11, *R^2^* = 0.75, *P* = 0.005

Beta-glucosidase: ln(*Z*) = 97.7(+ 15.6) (1/*RT_opt_*) – 49.9(+ 5.92), *N* = 11, *R^2^* = 0.81, *P* < 0.001

Beta-glucuronidase: ln(*Z*) = 63.7(+ 26.1) (1/*RT_opt_*) – 39.6(+ 9.82), *N* = 9, *R^2^* = 0.46, *P* = 0.049

1. *Substrate concentration*

Beta-galactosidase: ln(*A*) = -9.9(+ 19.4) (1/*RT_opt_*) – 8.4(+ 7.4), *N* = 13, *R^2^* = 0.02, *P* = 0.618

Alpha-amylase: ln(*A*) = -25.1(+ 15.8) (1/*RT_opt_*) – 11.8(+ 5.8), *N* = 11, *R^2^* = 0.22, *P* = 0.147

Beta-glucosidase: ln(*A*) = -22.9(+ 26.9) (1/*RT_opt_*) – 12.0(+ 10.2), *N* = 11, *R^2^* = 0.07, *P* = 0.412

Beta-glucuronidase: ln(*A*) = -20.4(+ 44.9) (1/*RT_opt_*) – 7.9(+ 16.9), *N* = 9, *R^2^* = 0.03, *P* = 0.662

**Supplementary Tables**

Table S1. ANOVA comparing activation energies (E_a_, kJ/mol) for process type, either diffusion or transport or product formation, and environment, either in *in vivo* versus *in vitro*.

Table S1. Two-Way ANOVA comparing activation energies (E_a_, kJ/mol) for process type, either diffusion or transport or product formation, and environment, either in *in vivo* versus *in vitro*.

|  | | | | | |
| --- | --- | --- | --- | --- | --- |
|  | | | | | |
| Source | SS | df |  | *F* | *P* |
| Corrected Model | 77893.52 | 11 |  | 13.941 | <0.0001 |
| Intercept | 104016.71 | 1 |  | 204.785 | <0.0001 |
| Process Type | 31978.73 | 2 |  | 15.740 | <0.0001 |
| Environment | 2799.78 | 2 |  | 2.756 | 0.068 |
| Type x Environment | 8126.71 | 5 |  | 3.200 | 0.010 |
| Error | 55364.56 | 109 |  |  |  |
| Total | 533610.17 | 121 |  |  |  |
| Corrected Total | 133258.09 | 120 |  |  |  |
| a. *R*^2^ = 0.585 (Adjusted *R*^2^ = 0.543) | | | | | |

Table S2. Analysis of Covariance comparing Arrhenius relationships between inverse of optimal temperature, 1/*RT_opt_* and reaction favorability, ln(*K_eq_*), enzyme catalytic capacity ln(*K_cat_*), and enzyme efficiency, ln(*K_cat_*/*K_m_*) for enzymes from organisms with different thermotolerance: thermophiles (Prokaryotes) and non-thermophiles (including both Eukaryotes and Prokaryotes)

|  | | | | | |
| --- | --- | --- | --- | --- | --- |
|  | | | | | |
| Source | SS | df |  | *F* | *P* |
| Corrected Model | 0.0343^a^ | 11 |  | 19.68 | <0.0001 |
| Intercept | 2.2148 | 1 |  | 14077.17 | <0.0001 |
| Thermotolerance | 0.0043 | 2 |  | 13.66 | <0.0001 |
| ln(*K_eq_*) | 0.0026 | 1 |  | 6.97 | 0.010 |
| ln(*K_cat_*) | 0.0011 | 1 |  | 2.43 | 0.122 |
| ln(*K_cat_*/*K_m_*) | 0.0002 | 1 |  | .030 | 0.871 |
| Thermotolerance x ln(*K_cat_*) | 0.0044 | 2 |  | 4.16 | <0.0001 |
| Thermotolerance x ln(*K_eq_*) | 0.0017 | 3 |  | 0.064 | 0.883 |
| Error | 0.0153 | 96 |  |  |  |
| Total | 14.7261 | 108 |  |  |  |
| Corrected Total | 0.0495 | 107 |  |  |  |
| *a R*^2^ = 0.693 (Adjusted *R*^2^ = 0.658) | | | | | |

| Table S3**.** Comparison of Arrhenius relationships between the inverse of optimal temperature (1/*RT_opt_*) with substrate concentration, ln(*A_o_*), and enzyme concentration, ln(Z) for four different enzymes (Enzyme). | | | | | |
| --- | --- | --- | --- | --- | --- |
|  | | | | | |
| Source | SS | df |  | *F* | *P* |
| Corrected Model | 0.0093^a^ | 11 |  | 8.989 | <0.0001 |
| Intercept | 0.0771 | 1 |  | 834.529 | <0.0001 |
| Enzyme | 0.0003 | 3 |  | 0.742 | 0.535 |
| ln(*A_o_*) | 0.0004 | 1 |  | 1.079 | 0.452 |
| ln(*Z*) | 0.0048 | 1 |  | 51.137 | <0.0001 |
| Enzyme x ln(*A_o_*) | 0.0009 | 3 |  | 1.934 | 0.149 |
| Enzyme x ln(*Z*) | 0.0011 | 3 |  | 2.253 | 0.103 |
| Error | 0.0034 | 30 |  |  |  |
| Total | 5.923 | 42 |  |  |  |
| Corrected Total | 0.012 | 41 |  |  |  |
| a. *R^2^* = 0.767 (Adjusted *R*^2^ = 0.682) | | | | | |

**References and Notes**

1. K. A. Dill, K. Ghosh, J. D. Schmitt, Physical limits of cells and proteomes. *Proceedings of the National Academy of Sciences* **108**, 17876–17882 (2011).

18. P. M. Kekenes-Huskey, C. E. Scott, S. Atalay, Quantifying the influence of the crowded cytoplasm on small molecule diffusion. *Journal of Physical Chemistry B* **120**, 8696-8706 (2016).

19. F. Roosen-Runge *et al.*, Protein self-diffusion in crowded solutions. *Proceedings of the National Academy of Sciences* **108**, 11815–11820 (2011).

35. R. Bujakiewicz-Koronska, J. Koronski, The principle of minimum entropy production for diffusion and heat transfer in open systems. *Czasopismo Techniczne* **122**, 23-35 (2011).

49. Griffith, S.M., Brewer, T.G., Steiner, J.J., Thermal dependence of the apparent *K_M_* of glutathione reductase from three wetland grasses and maize. Annals of Botany 87: 599-603 (2001).

50. K. J. Laidler, B. F. Peterman, in *Methods in Enzymology*. (Elsevier, New York, 1979), vol. 63, chap. 10, pp. 234-257.
